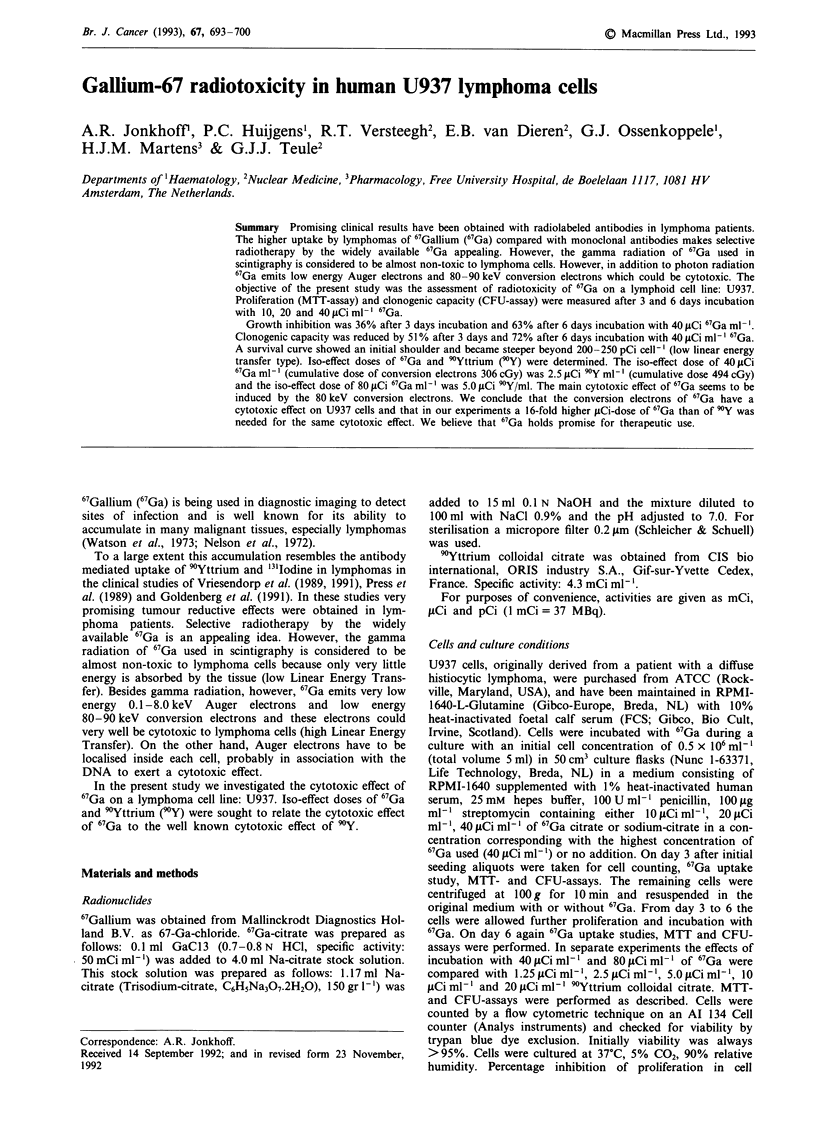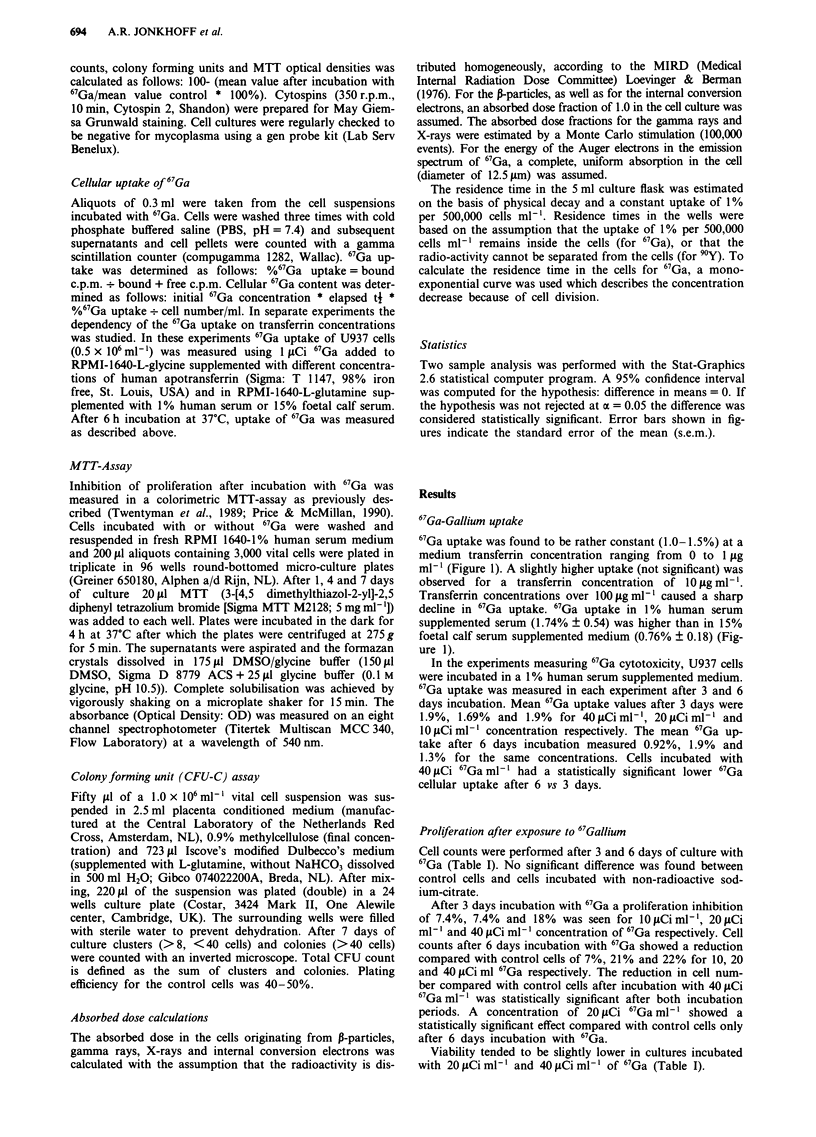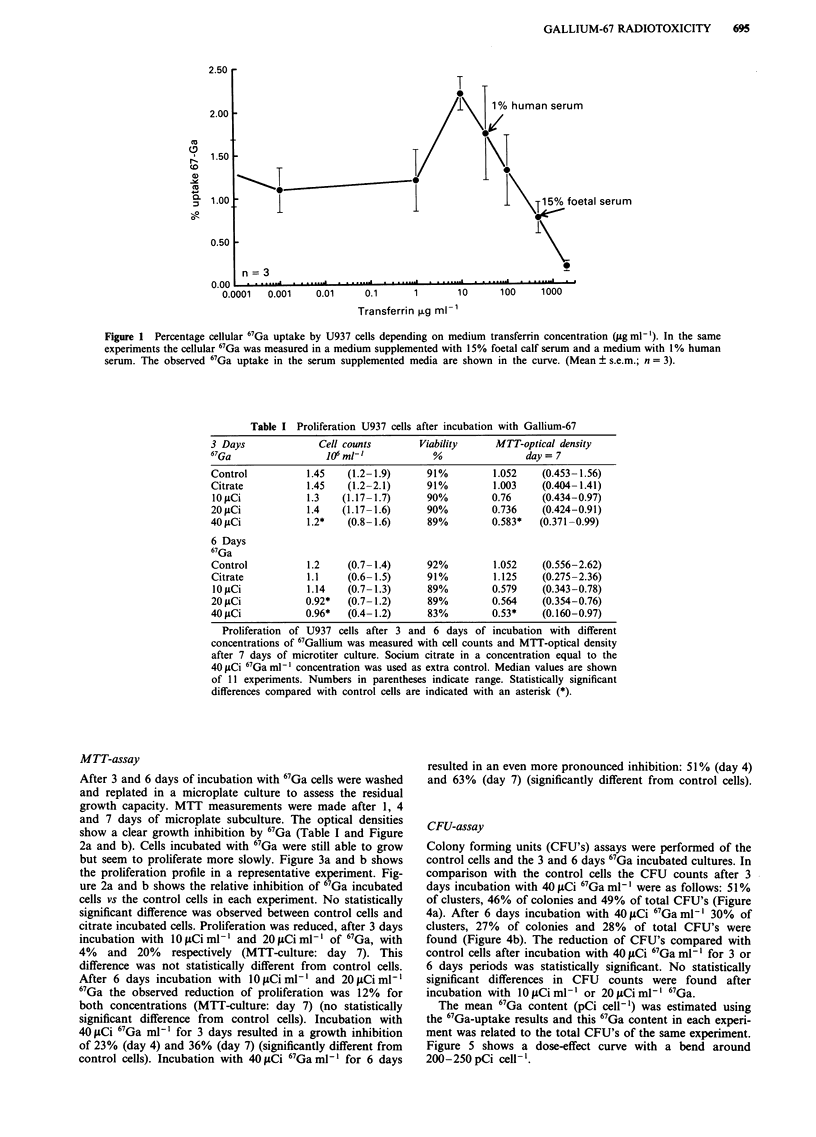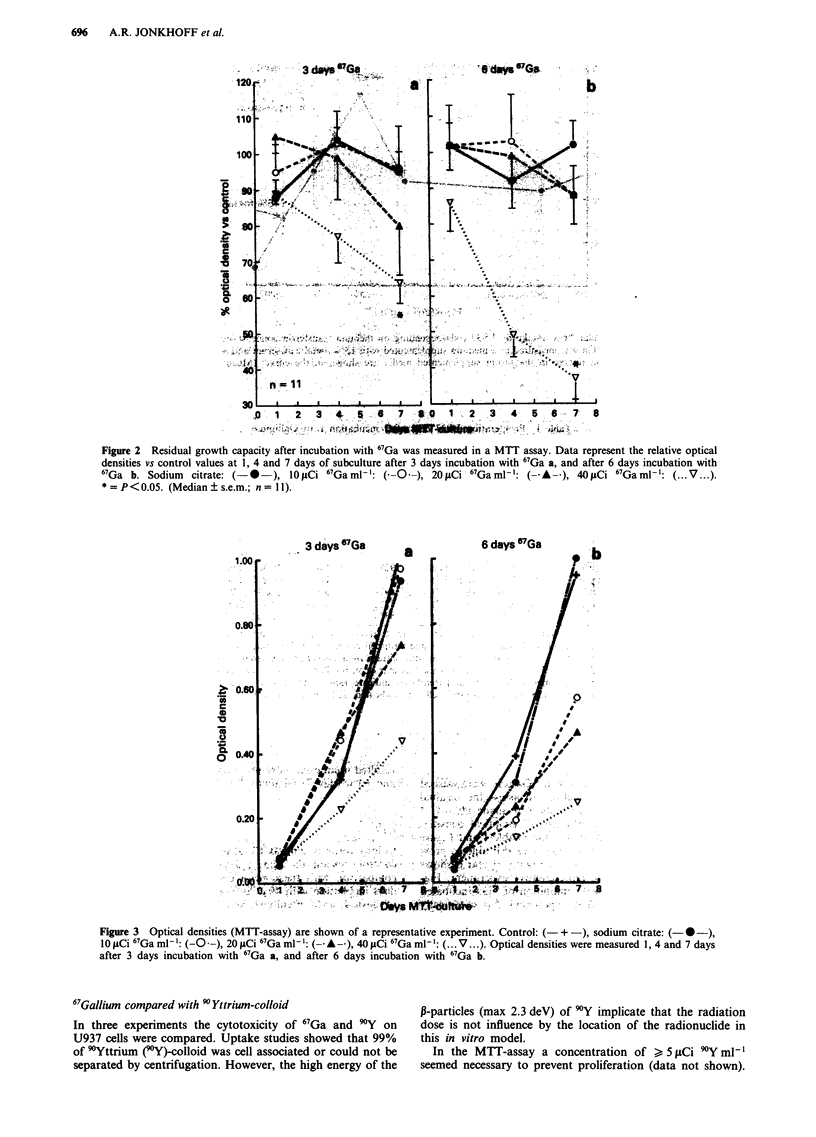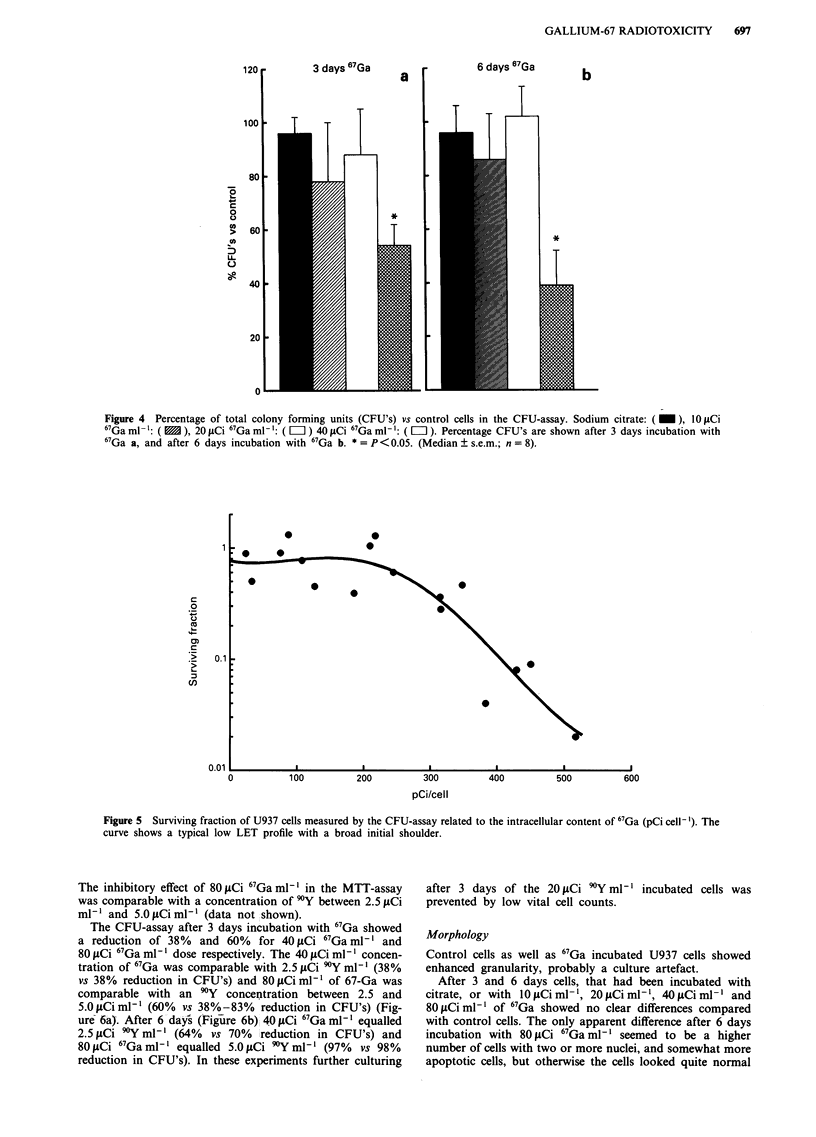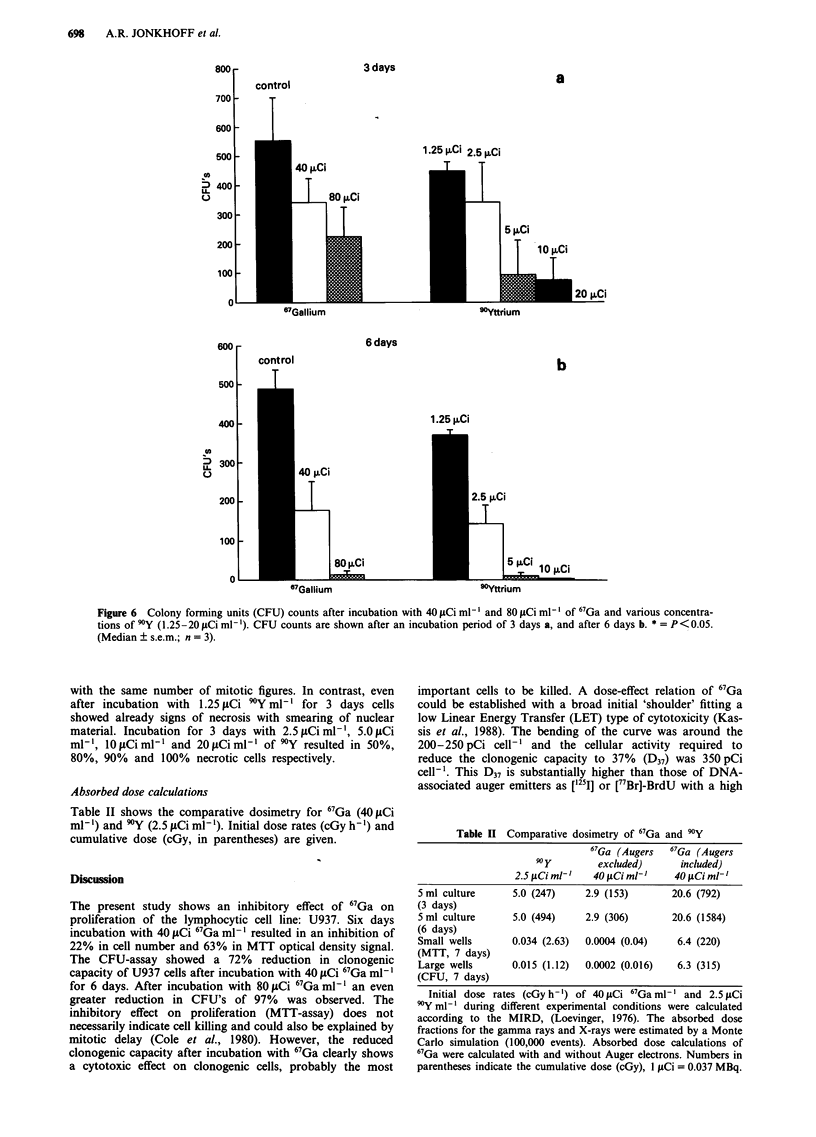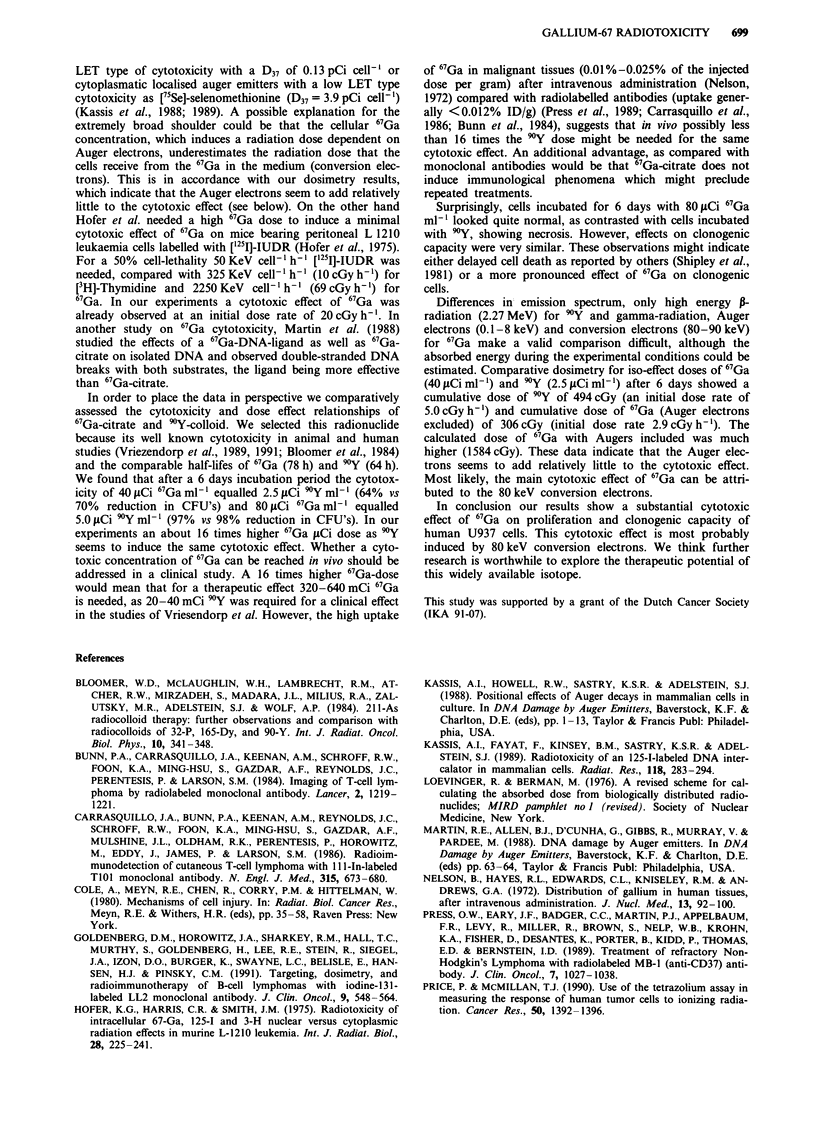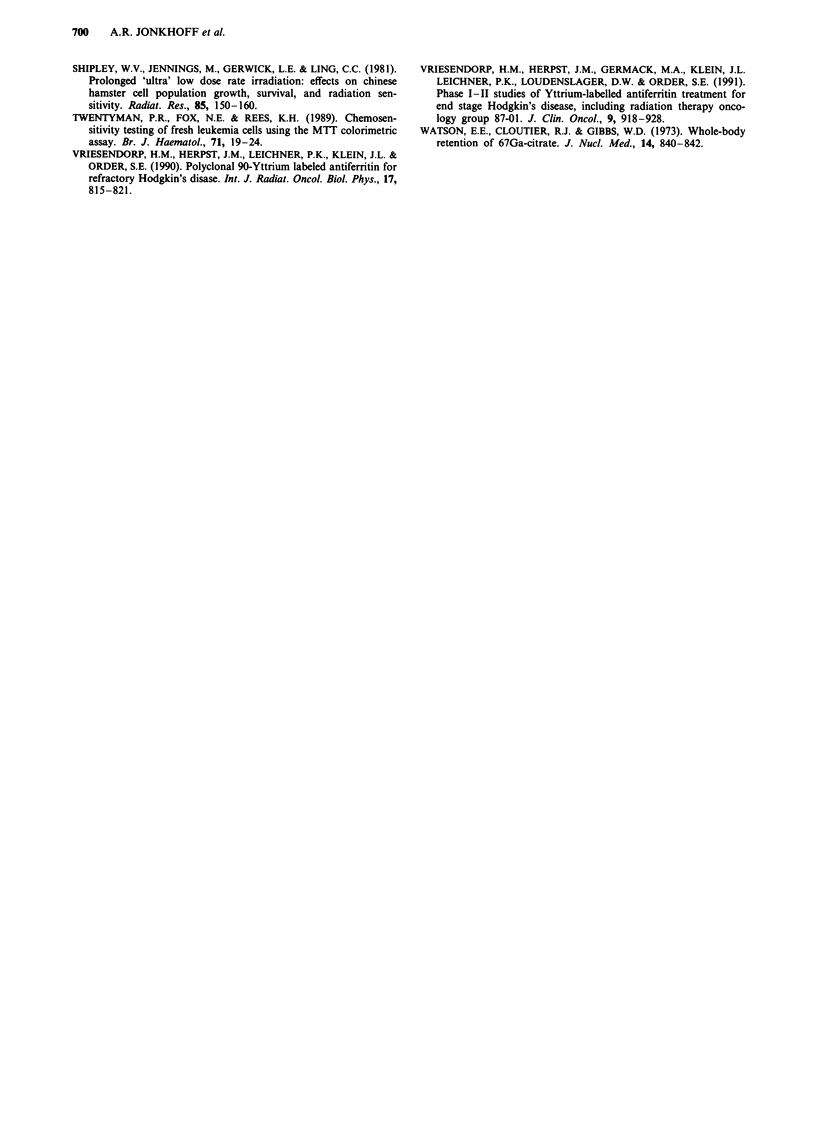# Gallium-67 radiotoxicity in human U937 lymphoma cells.

**DOI:** 10.1038/bjc.1993.128

**Published:** 1993-04

**Authors:** A. R. Jonkhoff, P. C. Huijgens, R. T. Versteegh, E. B. van Dieren, G. J. Ossenkoppele, H. J. Martens, G. J. Teule

**Affiliations:** Department of Haematology, Free University Hospital, Amsterdam, The Netherlands.

## Abstract

Promising clinical results have been obtained with radiolabeled antibodies in lymphoma patients. The higher uptake by lymphomas of 67Gallium (67Ga) compared with monoclonal antibodies makes selective radiotherapy by the widely available 67Ga appealing. However, the gamma radiation of 67Ga used in scintigraphy is considered to be almost non-toxic to lymphoma cells. However, in addition to photon radiation 67Ga emits low energy Auger electrons and 80-90 keV conversion electrons which could be cytotoxic. The objective of the present study was the assessment of radiotoxicity of 67Ga on a lymphoid cell line: U937. Proliferation (MTT-assay) and clonogenic capacity (CFU-assay) were measured after 3 and 6 days incubation with 10, 20 and 40 microCi ml-1 67Ga. Growth inhibition was 36% after 3 days incubation and 63% after 6 days incubation with 40 microCi 67Ga ml-1. Clonogenic capacity was reduced by 51% after 3 days and 72% after 6 days incubation with 40 microCi ml-1 67Ga. A survival curve showed an initial shoulder and became steeper beyond 200-250 pCi cell-1 (low linear energy transfer type). Iso-effect doses of 67Ga and 90Yttrium (90Y) were determined. The iso-effect dose of 40 microCi 67Ga ml-1 (cumulative dose of conversion electrons 306 cGy) was 2.5 microCi 90Y ml-1 (cumulative dose 494 cGy) and the iso-effect dose of 80 microCi 67Ga ml-1 was 5.0 microCi 90Y/ml. The main cytotoxic effect of 67Ga seems to be induced by the 80 keV conversion electrons. We conclude that the conversion electrons of 67Ga have a cytotoxic effect on U937 cells and that in our experiments a 16-fold higher microCi-dose of 67Ga than of 90Y was needed for the same cytotoxic effect. We believe that 67Ga holds promise for therapeutic use.


Br. J. Cancer (1993), 67, 693–700 © Macmillan Press Ltd., 1993

### Gallium-67 radiotoxicity in human U937 lymphoma cells

A.R. Jonkhoff[1], P.C. Huijgens[1], R.T. Versteegh[2], E.B. van Dieren[2], G.J. Ossenkoppele[1], H.J.M. Martens[3] & G.J.J. Teule[2]

*Departments of [1]Haematology, [2]Nuclear Medicine, [3]Pharmacology, Free University Hospital, de Boelelaan 1117, 1081 HV Amsterdam, The Netherlands.*

**Summary** Promising clinical results have been obtained with radiolabeled antibodies in lymphoma patients. The higher uptake by lymphomas of $^{67}$Gallium ($^{67}$Ga) compared with monoclonal antibodies makes selective radiotherapy by the widely available $^{67}$Ga appealing. However, the gamma radiation of $^{67}$Ga used in scintigraphy is considered to be almost non-toxic to lymphoma cells. However, in addition to photon radiation $^{67}$Ga emits low energy Auger electrons and 80–90 keV conversion electrons which could be cytotoxic. The objective of the present study was the assessment of radiotoxicity of $^{67}$Ga on a lymphoid cell line: U937. Proliferation (MTT-assay) and clonogenic capacity (CFU-assay) were measured after 3 and 6 days incubation with 10, 20 and 40 $\mu$Ci ml$^{-1}$ $^{67}$Ga.

Growth inhibition was 36% after 3 days incubation and 63% after 6 days incubation with 40 $\mu$Ci $^{67}$Ga ml$^{-1}$. Clonogenic capacity was reduced by 51% after 3 days and 72% after 6 days incubation with 40 $\mu$Ci ml$^{-1}$ $^{67}$Ga. A survival curve showed an initial shoulder and became steeper beyond 200–250 pCi cell$^{-1}$ (low linear energy transfer type). Iso-effect doses of $^{67}$Ga and $^{90}$Yttrium ($^{90}$Y) were determined. The iso-effect dose of 40 $\mu$Ci $^{67}$Ga ml$^{-1}$ (cumulative dose of conversion electrons 306 cGy) was 2.5 $\mu$Ci $^{90}$Y ml$^{-1}$ (cumulative dose 494 cGy) and the iso-effect dose of 80 $\mu$Ci $^{67}$Ga ml$^{-1}$ was 5.0 $\mu$Ci $^{90}$Y/ml. The main cytotoxic effect of $^{67}$Ga seems to be induced by the 80 keV conversion electrons. We conclude that the conversion electrons of $^{67}$Ga have a cytotoxic effect on U937 cells and that in our experiments a 16-fold higher $\mu$Ci-dose of $^{67}$Ga than of $^{90}$Y was needed for the same cytotoxic effect. We believe that $^{67}$Ga holds promise for therapeutic use.

$^{67}$Gallium ($^{67}$Ga) is being used in diagnostic imaging to detect sites of infection and is well known for its ability to accumulate in many malignant tissues, especially lymphomas (Watson *et al.*, 1973; Nelson *et al.*, 1972).

To a large extent this accumulation resembles the antibody mediated uptake of $^{90}$Yttrium and $^{131}$Iodine in lymphomas in the clinical studies of Vriesendorp *et al.* (1989, 1991), Press *et al.* (1989) and Goldenberg *et al.* (1991). In these studies very promising tumour reductive effects were obtained in lymphoma patients. Selective radiotherapy by the widely available $^{67}$Ga is an appealing idea. However, the gamma radiation of $^{67}$Ga used in scintigraphy is considered to be almost non-toxic to lymphoma cells because only very little energy is absorbed by the tissue (low Linear Energy Transfer). Besides gamma radiation, however, $^{67}$Ga emits very low energy 0.1–8.0 keV Auger electrons and low energy 80–90 keV conversion electrons and these electrons could very well be cytotoxic to lymphoma cells (high Linear Energy Transfer). On the other hand, Auger electrons have to be localised inside each cell, probably in association with the DNA to exert a cytotoxic effect.

In the present study we investigated the cytotoxic effect of $^{67}$Ga on a lymphoma cell line: U937. Iso-effect doses of $^{67}$Ga and $^{90}$Yttrium ($^{90}$Y) were sought to relate the cytotoxic effect of $^{67}$Ga to the well known cytotoxic effect of $^{90}$Y.

### Materials and methods

### *Radionuclides*

$^{67}$Gallium was obtained from Mallinckrodt Diagnostics Holland B.V. as 67-Ga-chloride. $^{67}$Ga-citrate was prepared as follows: 0.1 ml GaC13 (0.7–0.8 N HCl, specific activity: 50 mCi ml$^{-1}$) was added to 4.0 ml Na-citrate stock solution. This stock solution was prepared as follows: 1.17 ml Na-citrate (Trisodium-citrate, $C_6H_5Na_3O_7.2H_2O$), 150 gr l$^{-1}$) was added to 15 ml 0.1 N NaOH and the mixture diluted to 100 ml with NaCl 0.9% and the pH adjusted to 7.0. For sterilisation a micropore filter 0.2 $\mu$m (Schleicher & Schuell) was used.

$^{90}$Yttrium colloidal citrate was obtained from CIS bio international, ORIS industry S.A., Gif-sur-Yvette Cedex, France. Specific activity: 4.3 mCi ml$^{-1}$.

For purposes of convenience, activities are given as mCi, $\mu$Ci and pCi (1 mCi = 37 MBq).

### *Cells and culture conditions*

U937 cells, originally derived from a patient with a diffuse histiocytic lymphoma, were purchased from ATCC (Rockville, Maryland, USA), and have been maintained in RPMI-1640-L-Glutamine (Gibco-Europe, Breda, NL) with 10% heat-inactivated foetal calf serum (FCS; Gibco, Bio Cult, Irvine, Scotland). Cells were incubated with $^{67}$Ga during a culture with an initial cell concentration of 0.5 × 10$^6$ ml$^{-1}$ (total volume 5 ml) in 50 cm$^3$ culture flasks (Nunc 1-63371, Life Technology, Breda, NL) in a medium consisting of RPMI-1640 supplemented with 1% heat-inactivated human serum, 25 mM hepes buffer, 100 U ml$^{-1}$ penicillin, 100 $\mu$g ml$^{-1}$ streptomycin containing either 10 $\mu$Ci ml$^{-1}$, 20 $\mu$Ci ml$^{-1}$, 40 $\mu$Ci ml$^{-1}$ of $^{67}$Ga citrate or sodium-citrate in a concentration corresponding with the highest concentration of $^{67}$Ga used (40 $\mu$Ci ml$^{-1}$) or no addition. On day 3 after initial seeding aliquots were taken for cell counting, $^{67}$Ga uptake study, MTT- and CFU-assays. The remaining cells were centrifuged at 100 *g* for 10 min and resuspended in the original medium with or without $^{67}$Ga. From day 3 to 6 the cells were allowed further proliferation and incubation with $^{67}$Ga. On day 6 again $^{67}$Ga uptake studies, MTT and CFU-assays were performed. In separate experiments the effects of incubation with 40 $\mu$Ci ml$^{-1}$ and 80 $\mu$Ci ml$^{-1}$ of $^{67}$Ga were compared with 1.25 $\mu$Ci ml$^{-1}$, 2.5 $\mu$Ci ml$^{-1}$, 5.0 $\mu$Ci ml$^{-1}$, 10 $\mu$Ci ml$^{-1}$ and 20 $\mu$Ci ml$^{-1}$ $^{90}$Yttrium colloidal citrate. MTT- and CFU-assays were performed as described. Cells were counted by a flow cytometric technique on an AI 134 Cell counter (Analys instruments) and checked for viability by trypan blue dye exclusion. Initially viability was always >95%. Cells were cultured at 37°C, 5% $CO_2$, 90% relative humidity. Percentage inhibition of proliferation in cell

Correspondence: A.R. Jonkhoff.
Received 14 September 1992; and in revised form 23 November, 1992

counts, colony forming units and MTT optical densities was calculated as follows: 100- (mean value after incubation with $^{67}$Ga/mean value control * 100%). Cytospins (350 r.p.m., 10 min, Cytospin 2, Shandon) were prepared for May Giemsa Grunwald staining. Cell cultures were regularly checked to be negative for mycoplasma using a gen probe kit (Lab Serv Benelux).

*Cellular uptake of $^{67}$Ga*

Aliquots of 0.3 ml were taken from the cell suspensions incubated with $^{67}$Ga. Cells were washed three times with cold phosphate buffered saline (PBS, pH = 7.4) and subsequent supernatants and cell pellets were counted with a gamma scintillation counter (compugamma 1282, Wallac). $^{67}$Ga uptake was determined as follows: %$^{67}$Ga uptake = bound c.p.m. ÷ bound + free c.p.m. Cellular $^{67}$Ga content was determined as follows: initial $^{67}$Ga concentration * elapsed $t\frac{1}{2}$ * %$^{67}$Ga uptake ÷ cell number/ml. In separate experiments the dependency of the $^{67}$Ga uptake on transferrin concentrations was studied. In these experiments $^{67}$Ga uptake of U937 cells ($0.5 \times 10^6$ ml$^{-1}$) was measured using 1 μCi $^{67}$Ga added to RPMI-1640-L-glycine supplemented with different concentrations of human apotransferrin (Sigma: T 1147, 98% iron free, St. Louis, USA) and in RPMI-1640-L-glutamine supplemented with 1% human serum or 15% foetal calf serum. After 6 h incubation at 37°C, uptake of $^{67}$Ga was measured as described above.

*MTT-Assay*

Inhibition of proliferation after incubation with $^{67}$Ga was measured in a colorimetric MTT-assay as previously described (Twentyman *et al.*, 1989; Price & McMillan, 1990). Cells incubated with or without $^{67}$Ga were washed and resuspended in fresh RPMI 1640-1% human serum medium and 200 μl aliquots containing 3,000 vital cells were plated in triplicate in 96 wells round-bottomed micro-culture plates (Greiner 650180, Alphen a/d Rijn, NL). After 1, 4 and 7 days of culture 20 μl MTT (3-[4,5 dimethylthiazol-2-yl]-2,5 diphenyl tetrazolium bromide [Sigma MTT M2128; 5 mg ml$^{-1}$]) was added to each well. Plates were incubated in the dark for 4 h at 37°C after which the plates were centrifuged at 275 *g* for 5 min. The supernatants were aspirated and the formazan crystals dissolved in 175 μl DMSO/glycine buffer (150 μl DMSO, Sigma D 8779 ACS + 25 μl glycine buffer (0.1 M glycine, pH 10.5)). Complete solubilisation was achieved by vigorously shaking on a microplate shaker for 15 min. The absorbance (Optical Density: OD) was measured on an eight channel spectrophotometer (Titertek Multiscan MCC 340, Flow Laboratory) at a wavelength of 540 nm.

*Colony forming unit (CFU-C) assay*

Fifty μl of a $1.0 \times 10^6$ ml$^{-1}$ vital cell suspension was suspended in 2.5 ml placenta conditioned medium (manufactured at the Central Laboratory of the Netherlands Red Cross, Amsterdam, NL), 0.9% methylcellulose (final concentration) and 723 μl Iscove's modified Dulbecco's medium (supplemented with L-glutamine, without $NaHCO_3$ dissolved in 500 ml $H_2O$; Gibco 074022200A, Breda, NL). After mixing, 220 μl of the suspension was plated (double) in a 24 wells culture plate (Costar, 3424 Mark II, One Alewile center, Cambridge, UK). The surrounding wells were filled with sterile water to prevent dehydration. After 7 days of culture clusters (>8, <40 cells) and colonies (>40 cells) were counted with an inverted microscope. Total CFU count is defined as the sum of clusters and colonies. Plating efficiency for the control cells was 40–50%.

*Absorbed dose calculations*

The absorbed dose in the cells originating from β-particles, gamma rays, X-rays and internal conversion electrons was calculated with the assumption that the radioactivity is distributed homogeneously, according to the MIRD (Medical Internal Radiation Dose Committee) Loevinger & Berman (1976). For the β-particles, as well as for the internal conversion electrons, an absorbed dose fraction of 1.0 in the cell culture was assumed. The absorbed dose fractions for the gamma rays and X-rays were estimated by a Monte Carlo stimulation (100,000 events). For the energy of the Auger electrons in the emission spectrum of $^{67}$Ga, a complete, uniform absorption in the cell (diameter of 12.5 μm) was assumed.

The residence time in the 5 ml culture flask was estimated on the basis of physical decay and a constant uptake of 1% per 500,000 cells ml$^{-1}$. Residence times in the wells were based on the assumption that the uptake of 1% per 500,000 cells ml$^{-1}$ remains inside the cells (for $^{67}$Ga), or that the radio-activity cannot be separated from the cells (for $^{90}$Y). To calculate the residence time in the cells for $^{67}$Ga, a mono-exponential curve was used which describes the concentration decrease because of cell division.

*Statistics*

Two sample analysis was performed with the Stat-Graphics 2.6 statistical computer program. A 95% confidence interval was computed for the hypothesis: difference in means = 0. If the hypothesis was not rejected at $\alpha = 0.05$ the difference was considered statistically significant. Error bars shown in figures indicate the standard error of the mean (s.e.m.).

### Results

*$^{67}$Ga-Gallium uptake*

$^{67}$Ga uptake was found to be rather constant (1.0–1.5%) at a medium transferrin concentration ranging from 0 to 1 μg ml$^{-1}$ (Figure 1). A slightly higher uptake (not significant) was observed for a transferrin concentration of 10 μg ml$^{-1}$. Transferrin concentrations over 100 μg ml$^{-1}$ caused a sharp decline in $^{67}$Ga uptake. $^{67}$Ga uptake in 1% human serum supplemented serum (1.74% ± 0.54) was higher than in 15% foetal calf serum supplemented medium (0.76% ± 0.18) (Figure 1).

In the experiments measuring $^{67}$Ga cytotoxicity, U937 cells were incubated in a 1% human serum supplemented medium. $^{67}$Ga uptake was measured in each experiment after 3 and 6 days incubation. Mean $^{67}$Ga uptake values after 3 days were 1.9%, 1.69% and 1.9% for 40 μCi ml$^{-1}$, 20 μCi ml$^{-1}$ and 10 μCi ml$^{-1}$ concentration respectively. The mean $^{67}$Ga uptake after 6 days incubation measured 0.92%, 1.9% and 1.3% for the same concentrations. Cells incubated with 40 μCi $^{67}$Ga ml$^{-1}$ had a statistically significant lower $^{67}$Ga cellular uptake after 6 *vs* 3 days.

*Proliferation after exposure to $^{67}$Gallium*

Cell counts were performed after 3 and 6 days of culture with $^{67}$Ga (Table I). No significant difference was found between control cells and cells incubated with non-radioactive sodium-citrate.

After 3 days incubation with $^{67}$Ga a proliferation inhibition of 7.4%, 7.4% and 18% was seen for 10 μCi ml$^{-1}$, 20 μCi ml$^{-1}$ and 40 μCi ml$^{-1}$ concentration of $^{67}$Ga respectively. Cell counts after 6 days incubation with $^{67}$Ga showed a reduction compared with control cells of 7%, 21% and 22% for 10, 20 and 40 μCi ml $^{67}$Ga respectively. The reduction in cell number compared with control cells after incubation with 40 μCi $^{67}$Ga ml$^{-1}$ was statistically significant after both incubation periods. A concentration of 20 μCi $^{67}$Ga ml$^{-1}$ showed a statistically significant effect compared with control cells only after 6 days incubation with $^{67}$Ga.

Viability tended to be slightly lower in cultures incubated with 20 μCi ml$^{-1}$ and 40 μCi ml$^{-1}$ of $^{67}$Ga (Table I).

**Figure 1** Percentage cellular $^{67}$Ga uptake by U937 cells depending on medium transferrin concentration ($\mu$g ml$^{-1}$). In the same experiments the cellular $^{67}$Ga was measured in a medium supplemented with 15% foetal calf serum and a medium with 1% human serum. The observed $^{67}$Ga uptake in the serum supplemented media are shown in the curve. (Mean ± s.e.m.; $n = 3$).

**Table I** Proliferation U937 cells after incubation with Gallium-67

| *3 Days* *$^{67}$Ga* | *Cell counts* *$10^6$ ml$^{-1}$* | | *Viability* *%* | *MTT-optical density* *day = 7* | |
|---|---|---|---|---|---|
| Control | 1.45 | (1.2–1.9) | 91% | 1.052 | (0.453–1.56) |
| Citrate | 1.45 | (1.2–2.1) | 91% | 1.003 | (0.404–1.41) |
| 10 $\mu$Ci | 1.3 | (1.17–1.7) | 90% | 0.76 | (0.434–0.97) |
| 20 $\mu$Ci | 1.4 | (1.17–1.6) | 90% | 0.736 | (0.424–0.91) |
| 40 $\mu$Ci | 1.2* | (0.8–1.6) | 89% | 0.583* | (0.371–0.99) |
| 6 Days $^{67}$Ga | | | | | |
| Control | 1.2 | (0.7–1.4) | 92% | 1.052 | (0.556–2.62) |
| Citrate | 1.1 | (0.6–1.5) | 91% | 1.125 | (0.275–2.36) |
| 10 $\mu$Ci | 1.14 | (0.7–1.3) | 89% | 0.579 | (0.343–0.78) |
| 20 $\mu$Ci | 0.92* | (0.7–1.2) | 89% | 0.564 | (0.354–0.76) |
| 40 $\mu$Ci | 0.96* | (0.4–1.2) | 83% | 0.53* | (0.160–0.97) |

Proliferation of U937 cells after 3 and 6 days of incubation with different concentrations of $^{67}$Gallium was measured with cell counts and MTT-optical density after 7 days of microtiter culture. Socium citrate in a concentration equal to the 40 $\mu$Ci $^{67}$Ga ml$^{-1}$ concentration was used as extra control. Median values are shown of 11 experiments. Numbers in parentheses indicate range. Statistically significant differences compared with control cells are indicated with an asterisk (*).

*MTT-assay*

After 3 and 6 days of incubation with $^{67}$Ga cells were washed and replated in a microplate culture to assess the residual growth capacity. MTT measurements were made after 1, 4 and 7 days of microplate subculture. The optical densities show a clear growth inhibition by $^{67}$Ga (Table I and Figure 2a and b). Cells incubated with $^{67}$Ga were still able to grow but seem to proliferate more slowly. Figure 3a and b shows the proliferation profile in a representative experiment. Figure 2a and b shows the relative inhibition of $^{67}$Ga incubated cells *vs* the control cells in each experiment. No statistically significant difference was observed between control cells and citrate incubated cells. Proliferation was reduced, after 3 days incubation with 10 $\mu$Ci ml$^{-1}$ and 20 $\mu$Ci ml$^{-1}$ of $^{67}$Ga, with 4% and 20% respectively (MTT-culture: day 7). This difference was not statistically different from control cells. After 6 days incubation with 10 $\mu$Ci ml$^{-1}$ and 20 $\mu$Ci ml$^{-1}$ $^{67}$Ga the observed reduction of proliferation was 12% for both concentrations (MTT-culture: day 7) (no statistically significant difference from control cells). Incubation with 40 $\mu$Ci $^{67}$Ga ml$^{-1}$ for 3 days resulted in a growth inhibition of 23% (day 4) and 36% (day 7) (significantly different from control cells). Incubation with 40 $\mu$Ci $^{67}$Ga ml$^{-1}$ for 6 days resulted in an even more pronounced inhibition: 51% (day 4) and 63% (day 7) (significantly different from control cells).

*CFU-assay*

Colony forming units (CFU's) assays were performed of the control cells and the 3 and 6 days $^{67}$Ga incubated cultures. In comparison with the control cells the CFU counts after 3 days incubation with 40 $\mu$Ci $^{67}$Ga ml$^{-1}$ were as follows: 51% of clusters, 46% of colonies and 49% of total CFU's (Figure 4a). After 6 days incubation with 40 $\mu$Ci $^{67}$Ga ml$^{-1}$ 30% of clusters, 27% of colonies and 28% of total CFU's were found (Figure 4b). The reduction of CFU's compared with control cells after incubation with 40 $\mu$Ci $^{67}$Ga ml$^{-1}$ for 3 or 6 days periods was statistically significant. No statistically significant differences in CFU counts were found after incubation with 10 $\mu$Ci ml$^{-1}$ or 20 $\mu$Ci ml$^{-1}$ $^{67}$Ga.

The mean $^{67}$Ga content (pCi cell$^{-1}$) was estimated using the $^{67}$Ga-uptake results and this $^{67}$Ga content in each experiment was related to the total CFU's of the same experiment. Figure 5 shows a dose-effect curve with a bend around 200–250 pCi cell$^{-1}$.

**Figure 2** Residual growth capacity after incubation with $^{67}$Ga was measured in a MTT assay. Data represent the relative optical densities *vs* control values at 1, 4 and 7 days of subculture after 3 days incubation with $^{67}$Ga **a**, and after 6 days incubation with $^{67}$Ga **b**. Sodium citrate: (—●—), 10 μCi $^{67}$Ga ml$^{-1}$: (·–O·–), 20 μCi $^{67}$Ga ml$^{-1}$: (–·▲–·), 40 μCi $^{67}$Ga ml$^{-1}$: (...∇...). * = $P<0.05$. (Median ± s.e.m.; $n = 11$).

**Figure 3** Optical densities (MTT-assay) are shown of a representative experiment. Control: (— + —), sodium citrate: (—●—), 10 μCi $^{67}$Ga ml$^{-1}$: (–O·–), 20 μCi $^{67}$Ga ml$^{-1}$: (–·▲–·), 40 μCi $^{67}$Ga ml$^{-1}$: (...∇...). Optical densities were measured 1, 4 and 7 days after 3 days incubation with $^{67}$Ga **a**, and after 6 days incubation with $^{67}$Ga **b**.

*$^{67}$Gallium compared with $^{90}$Yttrium-colloid*

In three experiments the cytotoxicity of $^{67}$Ga and $^{90}$Y on U937 cells were compared. Uptake studies showed that 99% of $^{90}$Yttrium ($^{90}$Y)-colloid was cell associated or could not be separated by centrifugation. However, the high energy of the β-particles (max 2.3 deV) of $^{90}$Y implicate that the radiation dose is not influence by the location of the radionuclide in this *in vitro* model.

In the MTT-assay a concentration of ≥ 5 μCi $^{90}$Y ml$^{-1}$ seemed necessary to prevent proliferation (data not shown).

**Figure 4** Percentage of total colony forming units (CFU's) *vs* control cells in the CFU-assay. Sodium citrate: ( ■ ), 10 µCi $^{67}$Ga ml$^{-1}$: ( ▨ ), 20 µCi $^{67}$Ga ml$^{-1}$: ( ☐ ) 40 µCi $^{67}$Ga ml$^{-1}$: ( ☐ ). Percentage CFU's are shown after 3 days incubation with $^{67}$Ga **a**, and after 6 days incubation with $^{67}$Ga **b**. * = $P<0.05$. (Median ± s.e.m.; $n = 8$).

**Figure 5** Surviving fraction of U937 cells measured by the CFU-assay related to the intracellular content of $^{67}$Ga (pCi cell$^{-1}$). The curve shows a typical low LET profile with a broad initial shoulder.

The inhibitory effect of 80 µCi $^{67}$Ga ml$^{-1}$ in the MTT-assay was comparable with a concentration of $^{90}$Y between 2.5 µCi ml$^{-1}$ and 5.0 µCi ml$^{-1}$ (data not shown).

The CFU-assay after 3 days incubation with $^{67}$Ga showed a reduction of 38% and 60% for 40 µCi $^{67}$Ga ml$^{-1}$ and 80 µCi $^{67}$Ga ml$^{-1}$ dose respectively. The 40 µCi ml$^{-1}$ concentration of $^{67}$Ga was comparable with 2.5 µCi $^{90}$Y ml$^{-1}$ (38% *vs* 38% reduction in CFU's) and 80 µCi ml$^{-1}$ of 67-Ga was comparable with an $^{90}$Y concentration between 2.5 and 5.0 µCi ml$^{-1}$ (60% *vs* 38%–83% reduction in CFU's) (Figure 6a). After 6 days (Figure 6b) 40 µCi $^{67}$Ga ml$^{-1}$ equalled 2.5 µCi $^{90}$Y ml$^{-1}$ (64% *vs* 70% reduction in CFU's) and 80 µCi $^{67}$Ga ml$^{-1}$ equalled 5.0 µCi $^{90}$Y ml$^{-1}$ (97% *vs* 98% reduction in CFU's). In these experiments further culturing after 3 days of the 20 µCi $^{90}$Y ml$^{-1}$ incubated cells was prevented by low vital cell counts.

*Morphology*

Control cells as well as $^{67}$Ga incubated U937 cells showed enhanced granularity, probably a culture artefact.

After 3 and 6 days cells, that had been incubated with citrate, or with 10 µCi ml$^{-1}$, 20 µCi ml$^{-1}$, 40 µCi ml$^{-1}$ and 80 µCi ml$^{-1}$ of $^{67}$Ga showed no clear differences compared with control cells. The only apparent difference after 6 days incubation with 80 µCi $^{67}$Ga ml$^{-1}$ seemed to be a higher number of cells with two or more nuclei, and somewhat more apoptotic cells, but otherwise the cells looked quite normal

**Figure 6** Colony forming units (CFU) counts after incubation with 40 μCi $ml^{-1}$ and 80 μCi $ml^{-1}$ of $^{67}Ga$ and various concentrations of $^{90}Y$ (1.25–20 μCi $ml^{-1}$). CFU counts are shown after an incubation period of 3 days **a**, and after 6 days **b**. * = $P < 0.05$. (Median ± s.e.m.; $n = 3$).

with the same number of mitotic figures. In contrast, even after incubation with 1.25 μCi $^{90}Y\ ml^{-1}$ for 3 days cells showed already signs of necrosis with smearing of nuclear material. Incubation for 3 days with 2.5 μCi $ml^{-1}$, 5.0 μCi $ml^{-1}$, 10 μCi $ml^{-1}$ and 20 μCi $ml^{-1}$ of $^{90}Y$ resulted in 50%, 80%, 90% and 100% necrotic cells respectively.

*Absorbed dose calculations*

Table II shows the comparative dosimetry for $^{67}Ga$ (40 μCi $ml^{-1}$) and $^{90}Y$ (2.5 μCi $ml^{-1}$). Initial dose rates (cGy $h^{-1}$) and cumulative dose (cGy, in parentheses) are given.

### Discussion

The present study shows an inhibitory effect of $^{67}Ga$ on proliferation of the lymphocytic cell line: U937. Six days incubation with 40 μCi $^{67}Ga\ ml^{-1}$ resulted in an inhibition of 22% in cell number and 63% in MTT optical density signal. The CFU-assay showed a 72% reduction in clonogenic capacity of U937 cells after incubation with 40 μCi $^{67}Ga\ ml^{-1}$ for 6 days. After incubation with 80 μCi $^{67}Ga\ ml^{-1}$ an even greater reduction in CFU's of 97% was observed. The inhibitory effect on proliferation (MTT-assay) does not necessarily indicate cell killing and could also be explained by mitotic delay (Cole *et al.*, 1980). However, the reduced clonogenic capacity after incubation with $^{67}Ga$ clearly shows a cytotoxic effect on clonogenic cells, probably the most important cells to be killed. A dose-effect relation of $^{67}Ga$ could be established with a broad initial 'shoulder' fitting a low Linear Energy Transfer (LET) type of cytotoxicity (Kassis *et al.*, 1988). The bending of the curve was around the 200–250 pCi $cell^{-1}$ and the cellular activity required to reduce the clonogenic capacity to 37% ($D_{37}$) was 350 pCi $cell^{-1}$. This $D_{37}$ is substantially higher than those of DNA-associated auger emitters as [$^{125}I$] or [$^{77}Br$]-BrdU with a high

**Table II** Comparative dosimetry of $^{67}Ga$ and $^{90}Y$

| | *$^{90}Y$ 2.5 μCi $ml^{-1}$* | *$^{67}Ga$ (Augers excluded) 40 μCi $ml^{-1}$* | *$^{67}Ga$ (Augers included) 40 μCi $ml^{-1}$* |
|---|---|---|---|
| 5 ml culture (3 days) | 5.0 (247) | 2.9 (153) | 20.6 (792) |
| 5 ml culture (6 days) | 5.0 (494) | 2.9 (306) | 20.6 (1584) |
| Small wells (MTT, 7 days) | 0.034 (2.63) | 0.0004 (0.04) | 6.4 (220) |
| Large wells (CFU, 7 days) | 0.015 (1.12) | 0.0002 (0.016) | 6.3 (315) |

Initial dose rates (cGy $h^{-1}$) of 40 μCi $^{67}Ga\ ml^{-1}$ and 2.5 μCi $^{90}Y\ ml^{-1}$ during different experimental conditions were calculated according to the MIRD, (Loevinger, 1976). The absorbed dose fractions for the gamma rays and X-rays were estimated by a Monte Carlo simulation (100,000 events). Absorbed dose calculations of $^{67}Ga$ were calculated with and without Auger electrons. Numbers in parentheses indicate the cumulative dose (cGy), 1 μCi = 0.037 MBq.

LET type of cytotoxicity with a $D_{37}$ of 0.13 pCi cell$^{-1}$ or cytoplasmatic localised auger emitters with a low LET type cytotoxicity as [$^{75}$Se]-selenomethionine ($D_{37}$ = 3.9 pCi cell$^{-1}$) (Kassis *et al.*, 1988; 1989). A possible explanation for the extremely broad shoulder could be that the cellular $^{67}$Ga concentration, which induces a radiation dose dependent on Auger electrons, underestimates the radiation dose that the cells receive from the $^{67}$Ga in the medium (conversion electrons). This is in accordance with our dosimetry results, which indicate that the Auger electrons seem to add relatively little to the cytotoxic effect (see below). On the other hand Hofer *et al.* needed a high $^{67}$Ga dose to induce a minimal cytotoxic effect of $^{67}$Ga on mice bearing peritoneal L 1210 leukaemia cells labelled with [$^{125}$I]-IUDR (Hofer *et al.*, 1975). For a 50% cell-lethality 50 KeV cell$^{-1}$ h$^{-1}$ [$^{125}$I]-IUDR was needed, compared with 325 KeV cell$^{-1}$ h$^{-1}$ (10 cGy h$^{-1}$) for [$^{3}$H]-Thymidine and 2250 KeV cell$^{-1}$ h$^{-1}$ (69 cGy h$^{-1}$) for $^{67}$Ga. In our experiments a cytotoxic effect of $^{67}$Ga was already observed at an initial dose rate of 20 cGy h$^{-1}$. In another study on $^{67}$Ga cytotoxicity, Martin *et al.* (1988) studied the effects of a $^{67}$Ga-DNA-ligand as well as $^{67}$Ga-citrate on isolated DNA and observed double-stranded DNA breaks with both substrates, the ligand being more effective than $^{67}$Ga-citrate.

In order to place the data in perspective we comparatively assessed the cytotoxicity and dose effect relationships of $^{67}$Ga-citrate and $^{90}$Y-colloid. We selected this radionuclide because its well known cytotoxicity in animal and human studies (Vriezendorp *et al.*, 1989, 1991; Bloomer *et al.*, 1984) and the comparable half-lifes of $^{67}$Ga (78 h) and $^{90}$Y (64 h). We found that after a 6 days incubation period the cytotoxicity of 40 μCi $^{67}$Ga ml$^{-1}$ equalled 2.5 μCi $^{90}$Y ml$^{-1}$ (64% *vs* 70% reduction in CFU's) and 80 μCi $^{67}$Ga ml$^{-1}$ equalled 5.0 μCi $^{90}$Y ml$^{-1}$ (97% *vs* 98% reduction in CFU's). In our experiments an about 16 times higher $^{67}$Ga μCi dose as $^{90}$Y seems to induce the same cytotoxic effect. Whether a cytotoxic concentration of $^{67}$Ga can be reached *in vivo* should be addressed in a clinical study. A 16 times higher $^{67}$Ga-dose would mean that for a therapeutic effect 320–640 mCi $^{67}$Ga is needed, as 20–40 mCi $^{90}$Y was required for a clinical effect in the studies of Vriesendorp *et al.* However, the high uptake of $^{67}$Ga in malignant tissues (0.01%–0.025% of the injected dose per gram) after intravenous administration (Nelson, 1972) compared with radiolabelled antibodies (uptake generally <0.012% ID/g) (Press *et al.*, 1989; Carrasquillo *et al.*, 1986; Bunn *et al.*, 1984), suggests that *in vivo* possibly less than 16 times the $^{90}$Y dose might be needed for the same cytotoxic effect. An additional advantage, as compared with monoclonal antibodies would be that $^{67}$Ga-citrate does not induce immunological phenomena which might preclude repeated treatments.

Surprisingly, cells incubated for 6 days with 80 μCi $^{67}$Ga ml$^{-1}$ looked quite normal, as contrasted with cells incubated with $^{90}$Y, showing necrosis. However, effects on clonogenic capacity were very similar. These observations might indicate either delayed cell death as reported by others (Shipley *et al.*, 1981) or a more pronounced effect of $^{67}$Ga on clonogenic cells.

Differences in emission spectrum, only high energy β-radiation (2.27 MeV) for $^{90}$Y and gamma-radiation, Auger electrons (0.1–8 keV) and conversion electrons (80–90 keV) for $^{67}$Ga make a valid comparison difficult, although the absorbed energy during the experimental conditions could be estimated. Comparative dosimetry for iso-effect doses of $^{67}$Ga (40 μCi ml$^{-1}$) and $^{90}$Y (2.5 μCi ml$^{-1}$) after 6 days showed a cumulative dose of $^{90}$Y of 494 cGy (an initial dose rate of 5.0 cGy h$^{-1}$) and cumulative dose of $^{67}$Ga (Auger electrons excluded) of 306 cGy (initial dose rate 2.9 cGy h$^{-1}$). The calculated dose of $^{67}$Ga with Augers included was much higher (1584 cGy). These data indicate that the Auger electrons seems to add relatively little to the cytotoxic effect. Most likely, the main cytotoxic effect of $^{67}$Ga can be attributed to the 80 keV conversion electrons.

In conclusion our results show a substantial cytotoxic effect of $^{67}$Ga on proliferation and clonogenic capacity of human U937 cells. This cytotoxic effect is most probably induced by 80 keV conversion electrons. We think further research is worthwhile to explore the therapeutic potential of this widely available isotope.

This study was supported by a grant of the Dutch Cancer Society (IKA 91-07).

### References

BLOOMER, W.D., MCLAUGHLIN, W.H., LAMBRECHT, R.M., ATCHER, R.W., MIRZADEH, S., MADARA, J.L., MILIUS, R.A., ZALUTSKY, M.R., ADELSTEIN, S.J. & WOLF, A.P. (1984). 211-As radiocolloid therapy: further observations and comparison with radiocolloids of 32-P, 165-Dy, and 90-Y. *Int. J. Radiat. Oncol. Biol. Phys.*, **10**, 341–348.

BUNN, P.A., CARRASQUILLO, J.A., KEENAN, A.M., SCHROFF, R.W., FOON, K.A., MING-HSU, S., GAZDAR, A.F., REYNOLDS, J.C., PERENTESIS, P. & LARSON, S.M. (1984). Imaging of T-cell lymphoma by radiolabeled monoclonal antibody. *Lancer*, **2**, 1219–1221.

CARRASQUILLO, J.A., BUNN, P.A., KEENAN, A.M., REYNOLDS, J.C., SCHROFF, R.W., FOON, K.A., MING-HSU, S., GAZDAR, A.F., MULSHINE, J.L., OLDHAM, R.K., PERENTESIS, P., HOROWITZ, M., EDDY, J., JAMES, P. & LARSON, S.M. (1986). Radioimmunodetection of cutaneous T-cell lymphoma with 111-In-labeled T101 monoclonal antibody. *N. Engl. J. Med.*, **315**, 673–680.

COLE, A., MEYN, R.E., CHEN, R., CORRY, P.M. & HITTELMAN, W. (1980). Mechanisms of cell injury. In: *Radiat. Biol. Cancer Res.*, Meyn, R.E. & Withers, H.R. (eds), pp. 35–58, Raven Press: New York.

GOLDENBERG, D.M., HOROWITZ, J.A., SHARKEY, R.M., HALL, T.C., MURTHY, S., GOLDENBERG, H., LEE, R.E., STEIN, R., SIEGEL, J.A., IZON, D.O., BURGER, K., SWAYNE, L.C., BELISLE, E., HANSEN, H.J. & PINSKY, C.M. (1991). Targeting, dosimetry, and radioimmunotherapy of B-cell lymphomas with iodine-131-labeled LL2 monoclonal antibody. *J. Clin. Oncol.*, **9**, 548–564.

HOFER, K.G., HARRIS, C.R. & SMITH, J.M. (1975). Radiotoxicity of intracellular 67-Ga, 125-I and 3-H nuclear versus cytoplasmic radiation effects in murine L-1210 leukemia. *Int. J. Radiat. Biol.*, **28**, 225–241.

KASSIS, A.I., HOWELL, R.W., SASTRY, K.S.R. & ADELSTEIN, S.J. (1988). Positional effects of Auger decays in mammalian cells in culture. In *DNA Damage by Auger Emitters*, Baverstock, K.F. & Charlton, D.E. (eds), pp. 1–13, Taylor & Francis Publ: Philadelphia, USA.

KASSIS, A.I., FAYAT, F., KINSEY, B.M., SASTRY, K.S.R. & ADELSTEIN, S.J. (1989). Radiotoxicity of an 125-I-labeled DNA intercalator in mammalian cells. *Radiat. Res.*, **118**, 283–294.

LOEVINGER, R. & BERMAN, M. (1976). A revised scheme for calculating the absorbed dose from biologically distributed radionuclides; *MIRD pamphlet no 1 (revised)*. Society of Nuclear Medicine, New York.

MARTIN, R.E., ALLEN, B.J., D'CUNHA, G., GIBBS, R., MURRAY, V. & PARDEE, M. (1988). DNA damage by Auger emitters. In *DNA Damage by Auger Emitters*, Baverstock, K.F. & Charlton, D.E. (eds) pp. 63–64, Taylor & Francis Publ: Philadelphia, USA.

NELSON, B., HAYES, R.L., EDWARDS, C.L., KNISELEY, R.M. & ANDREWS, G.A. (1972). Distribution of gallium in human tissues, after intravenous administration. *J. Nucl. Med.*, **13**, 92–100.

PRESS, O.W., EARY, J.F., BADGER, C.C., MARTIN, P.J., APPELBAUM, F.R., LEVY, R., MILLER, R., BROWN, S., NELP, W.B., KROHN, K.A., FISHER, D., DESANTES, K., PORTER, B., KIDD, P., THOMAS, E.D. & BERNSTEIN, I.D. (1989). Treatment of refractory Non-Hodgkin's Lymphoma with radiolabeled MB-1 (anti-CD37) antibody. *J. Clin. Oncol.*, **7**, 1027–1038.

PRICE, P. & MCMILLAN, T.J. (1990). Use of the tetrazolium assay in measuring the response of human tumor cells to ionizing radiation. *Cancer Res.*, **50**, 1392–1396.

SHIPLEY, W.V., JENNINGS, M., GERWICK, L.E. & LING, C.C. (1981). Prolonged 'ultra' low dose rate irradiation: effects on chinese hamster cell population growth, survival, and radiation sensitivity. *Radiat. Res.*, **85**, 150–160.

TWENTYMAN, P.R., FOX, N.E. & REES, K.H. (1989). Chemosensitivity testing of fresh leukemia cells using the MTT colorimetric assay. *Br. J. Haematol.*, **71**, 19–24.

VRIESENDORP, H.M., HERPST, J.M., LEICHNER, P.K., KLEIN, J.L. & ORDER, S.E. (1990). Polyclonal 90-Yttrium labeled antiferritin for refractory Hodgkin's disase. *Int. J. Radiat. Oncol. Biol. Phys.*, **17**, 815–821.

VRIESENDORP, H.M., HERPST, J.M., GERMACK, M.A., KLEIN, J.L. LEICHNER, P.K., LOUDENSLAGER, D.W. & ORDER, S.E. (1991). Phase I–II studies of Yttrium-labelled antiferritin treatment for end stage Hodgkin's disease, including radiation therapy oncology group 87-01. *J. Clin. Oncol.*, **9**, 918–928.

WATSON, E.E., CLOUTIER, R.J. & GIBBS, W.D. (1973). Whole-body retention of 67Ga-citrate. *J. Nucl. Med.*, **14**, 840–842.